# A Curious Case of Hybrid Ameloblastoma

**DOI:** 10.7759/cureus.42512

**Published:** 2023-07-26

**Authors:** Tauseef A Sheikh, Priyanka P Kamble, Gargi Deshmukh, Akshit Vermani, Mohit Verma

**Affiliations:** 1 Oral and Maxillofacial Surgery, Aditya Dental College, Beed, IND; 2 Oral Pathology and Microbiology, Jawahar Medical Foundation's ACPM Dental College, Dhule, IND; 3 Dental Surgery, Deshmukh Dental Clinic, Aurangabad, IND; 4 Oral and Maxillofacial Surgery, Guru Nanak Dev Dental College, Sunam, IND

**Keywords:** aggressive, reoccurrence, surgical resection, hybrid tumor, ameloblastoma

## Abstract

Ameloblastoma is one of the most prevalent odontogenic tumors of epithelial origin, with several histological variations. However, among these variants, 'hybrid ameloblastoma' is infrequent and anomalous. The current case study demonstrates the existence of hybrid ameloblastoma in a 27-year-old female patient, which included desmoplastic, follicular, and acanthomatous patterns. The right side of the mandible was affected by tumor growth, with extensive bone involvement and neural invasion, resulting in a loss of sensation on that side. Although the tumor grows at a gradual pace, its enigmatic manifestation highlights the significance of a meticulous diagnosis. The course of treatment involved comprehensive resection of the tumor segment, followed by the recommended reconstructive surgery during the postoperative follow-up period.

## Introduction

Tooth development is an intricate process characterized by a series of multifaceted molecular interactions, which in turn may lead to the emergence of a distinctive array of odontogenic neoplasms and other pathologies exclusively localized to the jaw [1]. Odontogenic tumors (OTs) are a diverse and complex group of tumors exhibiting a broad spectrum of clinical and pathological features characterized by their exclusive occurrence in the jawbones and/or gingiva. These tumors, arising from the remnants of the tooth-forming apparatus or odontogenic epithelium, represent a significant diagnostic and therapeutic challenge owing to their varied presentation and unpredictable behavior [2]. Ameloblastoma, a benign OT, is a rare entity, accounting for almost 1% of all tumors and cysts that manifest in the jaw. However, it is the second most common OT after odontomas. This tumor has the potential to emerge at any stage of life; however, its prevalence is known to be highest during the third and fourth decades of life [3]. Radiologically, it can be discerned that this particular lesion presents itself as a conglomeration of mixed radiopaque and radiolucent materials with well-defined or ill-defined margins, which may exhibit either a unilocular or multilocular appearance, depending on the specific circumstances and characteristics of the lesion [4].

In 2005, the World Health Organization (WHO) classified ameloblastoma into four major histopathological types. These four types are solid/multicystic, extraosseous/peripheral, desmoplastic, and unicystic [5]. Furthermore, the predominant histopathological variants that are known to manifest in ameloblastoma are the dominant follicular and plexiform types, followed by acanthomatous and granular cell types [3]. Hybrid lesions, defined as those comprising two or more distinct areas that exhibit characteristic morphologic features of different entities, are a rarity in the literature. The definition, as posited by Ide et al., pertains to lesions that exhibit amalgamation of the histopathological characteristics of two or more neoplasms, as previously acknowledged [1]. The presence of these lesions poses a significant diagnostic challenge to dental practitioners. Takata et al. recently reported their findings regarding the prevalence of hybrid lesions in all ameloblastomas and concluded that the incidence of this phenomenon was 1.1% [6]. In 1987, Waldron and El-Mofty introduced a novel variant of ameloblastoma known as hybrid ameloblastoma. This variant is characterized by an unpredictable combination of histological features of desmoplastic ameloblastoma and conventional ameloblastoma [4]. This unique variant of ameloblastoma is particularly difficult to diagnose, highlighting the need for further research in this field. The objective of this report was to present an uncommon case to enhance the comprehension of this particular type of ameloblastic neoplasm, which may present challenges in both diagnosis and treatment owing to its diverse histopathological manifestations.

## Case presentation

**Figure 1:**
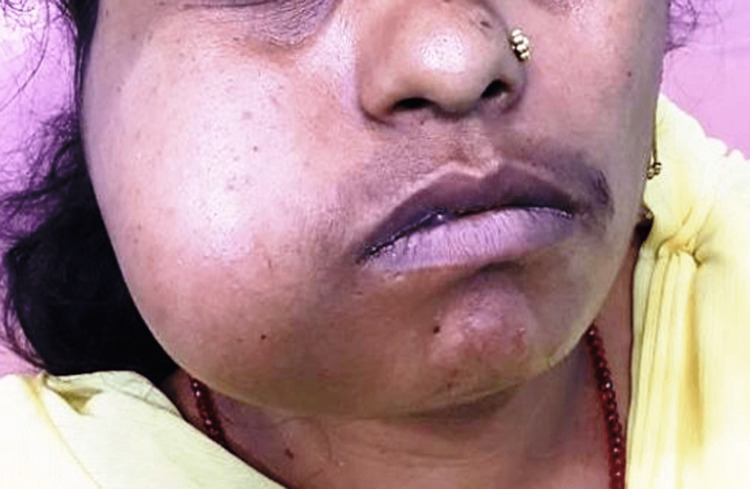
A diffuse swelling on the right side of the patient's face

A female patient aged 27 years reported to the Department of Oral and Maxillofacial Surgery in March 2023 with the chief complaint of pain and tenderness over the right side of the face for one year. The swelling started spontaneously and gradually increased in size up to its current magnitude. The patient's medical background was deemed noncontributory, signifying the absence of any noteworthy prior medical occurrences or circumstances that may have the potential to indicate the lesion. Additionally, following a thorough examination, it was established that there was no record of any physical harm or injury inflicted on the relevant anatomical location. Historical documentation indicates that the expansion was accompanied by a sensation deficiency on the affected side. Upon extraoral examination, unilateral asymmetry in the lower third of the face was observed. Furthermore, broad-based diffuse swelling was detected on the right side of the face, measuring approximately 4 cm × 6 cm and extending over the mandibular body, ramus, and condylar area. These findings were indicative of an intraosseous lesion that required thorough investigation and diagnosis (Figure 1).

Upon palpation, no discernible increase in temperature was observed in the affected region. Additionally, the swelling presented a hard consistency and was noncompressible and nonfluctuant. Furthermore, a distinct "crackling sound" was produced by pressurizing the area. Owing to the restrictive opening of the patient's mouth during the course of the disease, intraoral visibility was limited. The lesion extended from the lower right premolar region to the ramus area, with evident buccolingual bony expansion. Notably, the teeth in the affected region were found to be vital without mobility. A missing mandibular third molar was also observed.

**Figure 2:**
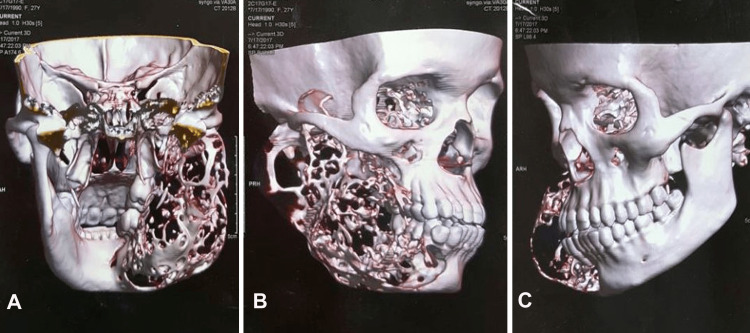
A CT scan showing infiltrative borders of the lesion A: Posterior view; B: Right side view; C: Left side view

A thorough evaluation was performed using fine-needle aspiration cytology (FNAC), in which an amber-colored fluid was detected, suggesting a potential diagnosis of a primordial cyst, residual cyst, or cystic ameloblastoma. A digital orthopantomogram (OPG) was acquired, and the results showed a clear multilocular radiolucency measuring approximately 7 cm × 5 cm. Radiolucency was ill-defined, extending anteriorly from the mesial side of tooth 43 up to 1 cm away from the border of the ramus of the mandible. Super-inferiorly, the radiopaque border extended from the sigmoid notch to the inferior border of the mandible. Both the mandibular condyle and the coronoid processes were involved. Axial CT revealed a sizable, expansile, destructive lesion of the mandible located on the right side of the mandible, extending up to the mandibular notch. Furthermore, thinning and expansion of the buccal and lingual cortical plates were observed (Figure 2).

**Figure 3:**
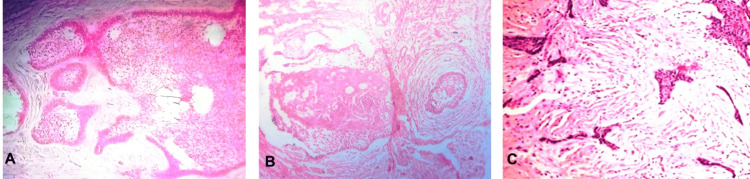
Histopathology showing odontogenic epithelium (H&E stained section at 40x magnification) A: Follicular form; B: Acanthomatous form; C: Desmoplastic form H&E: Hematoxylin & eosin

Involvement of the mandibular canal and mandibular nerve was also evident, along with the formation of septa. A probable diagnosis of aggressive ameloblastoma was established and confirmed using an incisional biopsy report from the Department of Oral Pathology. A biopsy was performed under local anesthesia, and upon histopathological examination, areas of small follicular islands of tumor cells of the odontogenic epithelium with peripheral ameloblasts and stellate reticulum-like cells were observed (Figure 3A). The stroma was collagenized and matured without calcification. Some follicles showed central acanthomatous changes (Figure 3B). The few borderline areas of the tumor stroma showed dense hyalinization with focal desmoplasia of the connective tissue as well as foci of squamous metaplasia infiltrating the soft tissues (Figure 3C). Abnormal mitosis and signs of malignancy were absent; however, local infiltration was predominant. The final histopathological picture was consistent with hybrid ameloblastoma.

The patient underwent broad segmental surgical excision under general anesthesia, but the surgery was not immediately followed by graft reconstruction because of extensive bony involvement of the mandibular nerve. The patient was continuously monitored and seated for reconstructive surgery during subsequent follow-up visits. Owing to the neural involvement, complete excision of the tumor proved to be arduous. Consequently, there was a high risk of recurrence. Thus, a follow-up regimen spanning a minimum of five years, followed by biennial appointments, was devised for our patient.

## Discussion

Ameloblastoma, a conventional odontogenic neoplasm, constitutes approximately 18% of all odontogenic jaw tumors. The most prevalent histological subtypes are follicular and plexiform, comprising 32.5% and 28.2% of cases, respectively, whereas desmoplastic is an exceedingly uncommon variant with incidence rates ranging from 4% to 13% [1,7]. Hybrid odontogenic lesions are a category of rare afflictions, with calcifying odontogenic cysts (COC) being the most frequently reported from a histopathological perspective. Calcifying odontogenic cysts are infrequent cysts that account for less than 1% of all odontogenic lesions. However, they are often found in conjunction with odontomas.

In the current case, a colossal hybrid ameloblastoma was observed in the posterior mandibular area of our 27-year-old female patient without any noteworthy medical history or prior trauma. The patient experienced pain, swelling, and loss of sensation on the affected side owing to neural involvement. According to Pontes et al., hybrid ameloblastomas are commonly found in individuals with an average age of 24.5 years and a male-to-female ratio of 0.9:1 [1]. Approximately 80% of all cases arise in the mandible, with 70% of cases observed in the ramus, as was evident in the present case.

In our case, the atypical hybrid ameloblastoma consisting of desmoplastic and classic variants exhibited follicular and acanthomatous patterns. However, this condition is extremely rare. According to Philipsen et al., a hybrid variant is an intermediate form of the desmoplastic subtype, possessing the microscopic characteristics of both the desmoplastic and classic subtypes. Radiographically, the desmoplastic variant presents features that resemble those of fibro-osseous diseases, as well as odontogenic cysts and tumors with mixed characteristics. This particular variant is considered an exceptionally uncommon entity, with incidence rates ranging from 4% to 13% [8]. The transformation of the primary desmoplastic variant into a conventional variant, or the occurrence of secondary desmoplastic changes in the stroma of the primary variant, remains uncertain. This adds to the mysterious and confusing nature of this variant. A few researchers have considered it a collision tumor [4]. Collision tumors, which originate from two distinct sites, are a unique phenomenon. One such example is hybrid ameloblastoma, which can be classified as a collision tumor because of the simultaneous occurrence of both desmoplastic and conventional variants.

Radiologically, hybrid variants manifest as areas of both radiopacity and radiolucency accompanied by irregular borders. Nevertheless, our case exhibited ill-defined radiolucency with a lattice arrangement of trabeculae, similar to conventional ameloblastoma in the mandible, as previously observed by Rai et al. in the anterior maxillae [4]. Histological examination of the current case revealed focused regions of desmoplasia at the borders of the lesion, showing a preponderance of squeezed epithelial islands. The other areas showed a follicular arrangement of the odontogenic epithelium with central stellate reticulum-like cells, squamous metaplasia, or microcyst formation. These findings align with the histological diagnostic standards outlined by Waldron and El-Mofty [9], who previously documented the existence of hybrid ameloblastomas featuring both desmoplastic and solid variants, which were also evident in the present case.

Accurate diagnosis of a lesion is crucial for patient management, regardless of lesion type. Nevertheless, the perplexing and ambiguous nature of hybrid ameloblastomas poses a challenge in terms of diagnosis and treatment [10]. There is no single method that can be universally applied to treat lesions of this type. The World Health Organization (WHO) recommends that large lesions be completely removed, whereas smaller lesions may be treated through enucleation and curettage. Nonetheless, the aggressive nature of hybrid ameloblastoma necessitates complete resection with broad margins to avoid recurrence, which must be followed by reconstruction of the bone defect [11]. The involvement of bony structures, in combination with their proximity to critical structures, presents a considerable challenge when attempting to achieve the complete resection of such tumors. Consequently, these tumors exhibit a high rate of recurrence [3]. Therefore, it is imperative to conduct long-term patient monitoring for five years, followed by biennial check-ups. This approach was implemented in the present study. After the patient underwent complete resection, she was subsequently referred for additional restorative surgical procedures, and her progress was consistently monitored through follow-up appointments.

## Conclusions

This study presents a unique example of a rare occurrence of hybrid ameloblastoma. The tumor demonstrated aggressive behavior, particularly in the posterior region of the mandible, and was found to involve vital structures such as the inferior alveolar nerve. The diagnosis was confirmed by careful analysis of the clinical, radiographic, and histological findings. Considering the high risk of recurrence associated with this tumor type, we decided to surgically excise the lesion and closely monitor the patient. The main objective of this case report is to raise awareness among clinicians about this rare condition and to emphasize the importance of prompt diagnosis and management of the lesion.
